# Monitoring Biosensor Activity in Living Cells with Fluorescence Lifetime Imaging Microscopy

**DOI:** 10.3390/ijms131114385

**Published:** 2012-11-07

**Authors:** Julia M. Hum, Amanda P. Siegel, Fredrick M. Pavalko, Richard N. Day

**Affiliations:** Department of Cellular and Integrative Physiology, Indiana University School of Medicine, 635 Barnhill Dr., MS 333, Indianapolis, IN 46202, USA; E-Mails: jhum@iupui.edu (J.M.H.); apsiegel@iupui.edu (A.P.S.); fpavalko@iupui.edu (F.M.P.)

**Keywords:** fluorescent protein, biosensor probe, fluorescence resonance energy transfer (FRET), fluorescence lifetime imaging microscopy (FLIM), cell signaling

## Abstract

Live-cell microscopy is now routinely used to monitor the activities of the genetically encoded biosensor proteins that are designed to directly measure specific cell signaling events inside cells, tissues, or organisms. Most fluorescent biosensor proteins rely on Förster resonance energy transfer (FRET) to report conformational changes in the protein that occur in response to signaling events, and this is commonly measured with intensity-based ratiometric imaging methods. An alternative method for monitoring the activities of the FRET-based biosensor proteins is fluorescence lifetime imaging microscopy (FLIM). FLIM measurements are made in the time domain, and are not affected by factors that commonly limit intensity measurements. In this review, we describe the use of the digital frequency domain (FD) FLIM method for the analysis of FRET signals. We illustrate the methods necessary for the calibration of the FD FLIM system, and demonstrate the analysis of data obtained from cells expressing “FRET standard” fusion proteins. We then use the FLIM-FRET approach to monitor the changes in activities of two different biosensor proteins in specific regions of single living cells. Importantly, the factors required for the accurate determination and reproducibility of lifetime measurements are described in detail.

## 1. Introduction

The use of microscopy to monitor fluorescent reporters that “sense” specific subcellular events inside single living cells are providing an unprecedented view of the spatial and temporal regulation of cell signaling events. These genetically encoded fluorescent reporters, known as biosensor proteins, combine sensing units to detect specific cell signaling events with reporter modules that incorporate the fluorescent proteins (FPs). The evolution of the newer generations of FPs with optimized characteristics has improved the detection of signals from the biosensor proteins expressed inside living cells [1]. This has given cell biologists access to an impressive collection of molecular tools to investigate the spatial and temporal regulation of cell signaling events.

Many different design strategies have been used to develop the genetically encoded biosensor proteins, but most rely on Förster resonance energy transfer (FRET) microscopy to report the changes in protein conformation that accompany the targeted signaling event [2–6]. FRET is a process through which energy absorbed by one fluorophore (the “donor”) is transferred directly to another nearby molecule (the “acceptor”) via a nonradiative pathway. This transfer of energy depletes the donor’s excited-state energy, quenching its fluorescence emission, while causing increased emission from a fluorescent acceptor. Since the distance over which efficient energy transfer can occur is limited to less than 80 Å, FRET can be used monitor dynamic conformational changes in biosensor proteins that occur in response to signaling events inside living cells, tissues, and organisms [5–9].

There are a variety of ways to detect changes in the FRET signals from biosensor proteins that are expressed inside living cells [7]. The most accurate methods, however, are those that detect the changes in the donor fluorescent lifetime that occur because of energy transfer [10]. Here, we review how fluorescence lifetime imaging microscopy (FLIM) is used to measure the changing FRET signals from biosensor probes inside living cells. The approach is first demonstrated by FRET measurements from protein standards designed to validate microscope systems for the detection of FRET [11]. The frequency domain (FD) FLIM method is then used to characterize the activity of a Src-biosensor inside living bone cells in response to a mechanical stimulus applied by dynamic fluid flow over the cell surface, and finally to evaluate cAMP activity within pituitary cells in response to an agonist.

## 2. The FP-Based Biosensors

**Figure 1:**
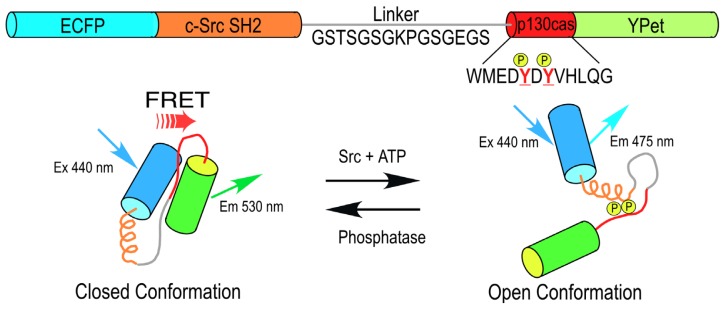
A schematic diagram of the Src biosensor (adapted from [19]).

The modular design of the sensing and reporter units of the genetically encoded biosensor proteins has enabled the development of probes to detect a wide variety of cellular events. Generally, the reporter unit consists of a pair of FPs that share the significant spectral overlap required for efficient FRET [12]. The sensing unit serves as a linker between the FPs, and includes an element that is modified by the targeted biological event, and a binding motif that recognizes the modification (for example, see Figure 1). This allows the sensing unit to change its conformation in response to a specific signaling event, altering the distance between the FP pair. The changing intramolecular FRET signal from these single chain biosensor proteins can be monitored in real time, allowing measurement of the spatiotemporal dynamics of protein activities [2–6]. However, signaling events (e.g., phosphorylation) may only be part of the activation dynamics, and the direct binding to other proteins may also be required. This can be detected by dual chain biosensors designed to detect intermolecular FRET that occurs when the separate donor- and the acceptor-labeled proteins interact. In this situation, FLIM can be used to detect the populations of unquenched donors and donors quenched by FRET [7,10]. In contrast, traditional approaches to detect these events, such as the use of phosphorylation-specific antibodies and immunochemical staining, involve the fixation of the cells or tissues, and have limited utility for reporting the dynamics of these events.

These genetically encoded reporters are easily transferred to cells in culture, and can be directed into specific subcellular organelles by incorporating suitable targeting sequences. Furthermore, the transgenes encoding these probes have been successfully introduced into a variety of organisms. For example, calcium sensing biosensor proteins have been used to image biological activities in living *C. elegans*, *Drosophila*, and Zebrafish [13–15]. The imaging of biosensor activities in transgenic mice, however, has proven problematic, and this may be related to the low-level expression of the proteins caused by transgene silencing [16]. Recently, a transposon-mediated gene transfer method was used to generate transgenic mice with a high-level of biosensor expression. This allowed the activity of these reporters to be monitored in several different tissues by two-photon excitation microscopy [17].

An important advantage of these genetically encoded probes is that they not only report the dynamic activity over time, but also provide critical information about the spatial compartmentalization of the signaling events. For example, the non-receptor tyrosine kinase Src plays a central role in a variety of cellular functions, including changes in cell morphology, cell migration, and processes linked to angiogenesis and cancer. A FRET-based Src biosensor protein was developed by Wang *et al.*[18,19] that uses cyan FP (CFP) and yellow FP (YFP) variants in the reporter unit adjoining a sensor unit consisting of the c-Src substrate from p130 cas that is linked to the c-Src SH2 domain (Figure 1). This single chain biosensor protein is in a “closed conformation” before phosphorylation of the p130 cas substrate, yielding a high FRET efficiency. The phosphorylation of the substrate peptide allows binding to the SH2 domain, which induces a conformational change that separates the FPs of the reporting unit, reducing the FRET signal (Figure 1). Studies using this probe revealed that Src activity is differentially regulated by cytoskeletal components within different compartments in the plasma membrane [20].

**Figure 2:**
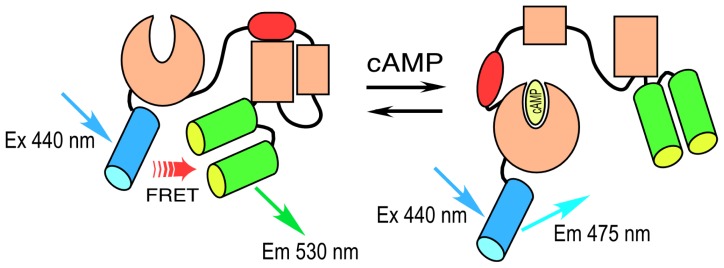
A schematic diagram of the cAMP biosensor based on the Epac protein (adapted from [23]).

A different strategy was used to develop a cAMP-responsive biosensor probe. Here, instead of engineering a sensor unit, investigators took advantage of the cAMP-dependent conformational change that occurs in the “exchange proteins activated by cAMP” (Epacs) to develop a sensor peptide. Epacs are cAMP receptor protein-mediated guanine nucleotide exchange factors that contain a cAMP-binding site in their *N*-terminal regulatory domain. Epac is folded in an inactive conformation at low cAMP levels, but responds to increasing cAMP binding by unfolding. A single-chain FRET probe to detect changing levels of intracellular cAMP was developed by sandwiching the Epac protein between CFP and YFP variants [21,22]. The binding of cAMP to the Epac sensor unit drives a large conformational change in the protein. This causes the separation of the reporter unit FPs, leading to a decrease in FRET and the coincident increase in the donor lifetime. An improved version of this biosensor protein was recently developed, called T_EPAC_VV, that uses monomeric (m) Turquoise, a bright variant of CFP, as the donor for a pair of optimized YFP acceptors [23] (Figure 2).

The Epac-based biosensor probes can report the spatiotemporal dynamics of cAMP inside single living cells, but do not measure the signaling cascade that is activated by the rise in intracellular cAMP. The mediator of cAMP activity within the cell is protein kinase A (PKA). Here, a different biosensor probe, the protein kinase A activity reporter (AKAR), was developed that contains a sensor unit that undergoes a conformational change in response to phosphorylation by PKA. This biosensor reports the PKA-dependent signaling that occurs in response to a rise in intracellular cAMP. Importantly, the AKAR reporter was used simultaneously with the Epac-based biosensor to examine the temporal correlation of cAMP dynamics and PKA activation within the same living cells [22,24].

The changing signal from the biosensor proteins is most commonly detected by ratiometric methods that monitor the quenching of the donor signal relative to the increase in the acceptor signal resulting from FRET (e.g., see [22,24]). The distinct advantage of this approach is speed. Ratiometric imaging can report the changes in biosensor protein activities on the time scale of seconds. However, most of the current biosensor probes have a very limited dynamic range (the difference between the minimum and maximum signal), and this can be a major disadvantage for ratiometric imaging. Furthermore, while it is important to select the reporter unit donor and acceptor FPs with an optimal spectral overlap to allow FRET, ratiometric imaging requires FPs with sufficient spectral separation in their peak excitation and emission to avoid cross-excitation and signal bleedthrough [6]. The reporter units in the most widely used FRET-based biosensors use CFP and YFP variants, and the direct excitation of YFP at the CFP excitation wavelength, coupled with the CFP signal bleed-through into the acceptor channel, collectively known as signal crosstalk, can be problematic. Alternative approaches avoid the problem of signal crosstalk by measuring the effect of FRET on the donor fluorophore alone, and this is what FLIM does.

## 3. Fluorescence Lifetime Imaging Microscopy

The fluorescence lifetime is the average time that a fluorophore spends in the excited state before returning to the ground state; an event generally coupled to the emission of a photon. The fluorescence lifetime is a photophysical property of every fluorophore, and most fluorescent molecules used for biological studies have lifetimes ranging between one to about ten nanoseconds (ns). The accurate measurement of changes in the fluorescence lifetime can report events in the local environments surrounding the fluorophore that alter its photophysical behavior. For example, energy transfer is a quenching process that depopulates the excited state of the donor fluorophore. When FRET occurs, there will be a shortening of the donor lifetime, which can be quantified by FLIM [10,25–27].

FLIM is one of the most accurate approaches for quantifying FRET signals because the measurements are made only in the donor channel so signal crosstalk is eliminated by selecting a donor channel emission bandwidth that avoids the acceptor emission [7,10]. This makes the FLIM approach the most direct method for monitoring the conformational changes in biosensor probes in response to signaling events. Moreover, it enables the mapping of the spatial distribution of probe lifetimes inside living cells. FLIM is especially useful for imaging biological samples, since it is a measurement of time that is not affected by variations in the probe concentration, excitation intensity, light scatter, and other factors that can limit intensity-based measurements. The FLIM methodologies have evolved significantly in recent years, and now encompass the materials science, as well as biomedical research applications, and clinical assay development [10]. The FLIM techniques are broadly subdivided into the time domain and the frequency domain (FD) methods. The physics that underlies these two different methods is identical, but they differ in the way the measurements are analyzed [28]. Here, we review the use of the FD FLIM method to measure FRET from biosensor probes.

### 3.1. The Analysis of FD FLIM Measurements

**Figure 3:**
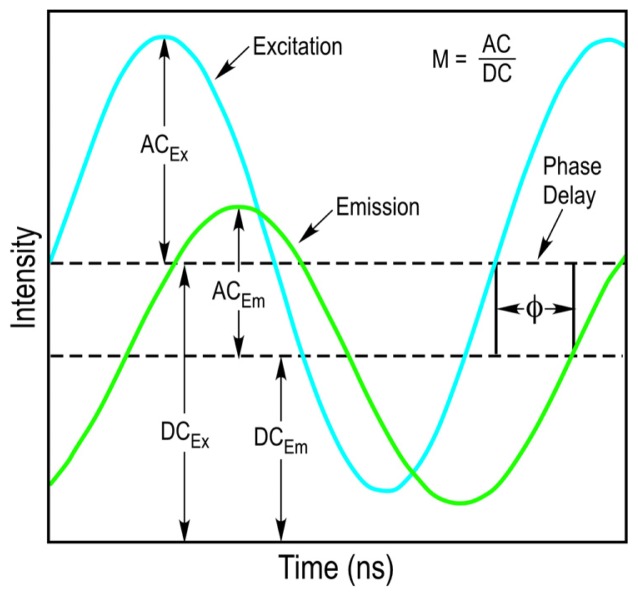
Frequency domain fluorescence lifetime imaging microscopy (FD FLIM) measurements. Excitation for FD FLIM is achieved using a light source that is modulated at high radio frequencies. The emission signals from the specimen are detected and analyzed for the phase delay (*Φ*) and changes in the modulation ratio (*M* = *AC*/*DC*) relative to that of the excitation source. The sine-cosine transforms of these frequency characteristics are directly plotted using the phasor coordinates to determine the fluorescence lifetime at each image pixel (see text for details).

The FD FLIM approach uses a light source that is modulated at high radio frequencies to excite the fluorophores in an experimental sample. The waveform of the excitation light source does not need to be sinusoidal and pulsed sources, such as those employed in two-photon excitation can also be used [10]. The fundamental modulation frequency is selected based on the lifetime of the fluorophore that is used, and usually ranges between 10 to 200 megahertz (MHz) for the measurement of nanosecond decays. Since the light source illuminating the sample is modulated, the emission signal from the sample will also be modulated. However, because of the fluorescence lifetime of the fluorophore, there will be a delay in the phase and a change in the modulation ratio of the emission signal relative to the excitation source (Figure 3). The FD FLIM approach compares the waveforms of the excitation source and the emission signal, and analyzes the changes in the phase and the modulation ratio to extract the fluorescence lifetime of the fluorophore at each pixel of an image.

The measurements obtained with FD FLIM are commonly analyzed using the phasor plot method [29–32]. This method was originally developed as a way to analyze transient responses to repetitive perturbations, and can be applied to any system with frequency characteristics. The phasor plot is a global representation of the relative modulation fraction (*M*) and the phase delay (*Φ*) of the emission signal from every pixel in an image, allowing the direct determination of the fluorescence lifetime of a fluorophore. The sine-cosine transforms of the emission signal frequency characteristics at each image pixel are represented by the phasor coordinates:

$$\begin{array}{l} x-\text{axis}:G=M(\omega )\times \text{cos}\;\Phi (\omega )\\ y-\text{axis}:S=M(\omega )\times \text{sin}\;\Phi (\omega ) \end{array}$$

where *ω* is the frequency of excitation. A lifetime vector is plotted for each image pixel, where *M* is the distance from the origin and *Φ* is the angle from the *x*-axis. For pixels with a single lifetime component, the lifetime determined by *Φ* and the lifetime determined by *M* will be equal. In the coordinate system these points will fall on a “universal semicircle” with longer lifetimes to the left (0, 0 is infinite lifetime) and shorter lifetimes to right [30]. In contrast, populations of fluorophores involved in FRET will have multiple lifetime components, and the distribution of points will fall inside the semicircle [32]. Most important, the polar plot does not require a fitting model to determine fluorescence lifetime distributions, but rather expresses the overall lifetime at each pixel in terms of the phasor coordinates. This approach can also be used to analyze FLIM data acquired by the time domain method, where the signals are first transformed to the frequency domain prior to analysis.

### 3.2. The Calibration of a FD FLIM System

Before imaging experimental samples, it is necessary to calibrate the FLIM system using a fluorescence lifetime standard. The fluorescence lifetimes for many different fluorophores have been established under standard conditions, and these can be used for calibration of a FLIM system [33]. It is important, however, to choose a standard fluorophore with excitation and emission properties that are similar to the fluorophore that is used in the experimental samples. For example, the dye Coumarin 6 dissolved in ethanol has a peak excitation of 460 nm and a peak emission of 505 nm, and a reference lifetime of 2.5 ns. It is often used as a calibration standard for the variants of CFP, which have similar spectral characteristics and lifetimes between 2.5 and 4.5 ns.

**Figure 4:**
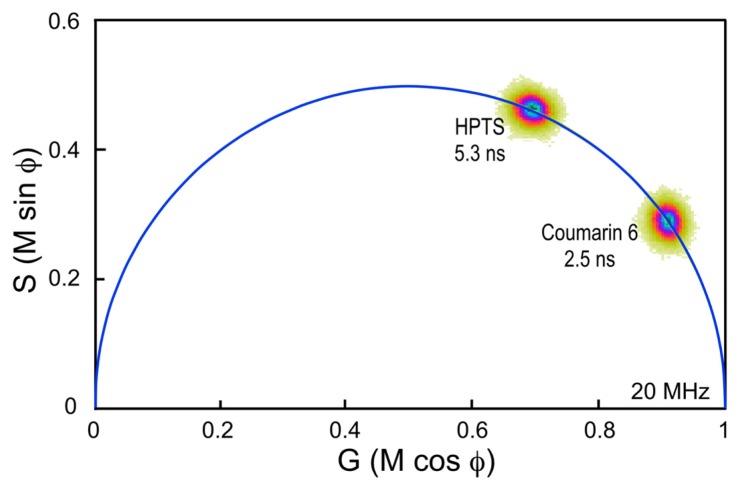
The phasor plot analysis of Coumarin 6 and HPTS measured at 20 MHz. The frequency characteristics for each pixel in the 256 × 256 pixel image (65,536 points) are displayed as described in Section 3.1. A threshold is applied to eliminate background noise, and a Gaussian 2D spatial convolution filter is used for smoothing. The distribution for Coumarin 6 falls on the universal semicircle, representing a single exponential lifetime of 2.5 ns. The phasor distribution for HPTS is shifted to the left along the semicircle, representing the longer lifetime of 5.3 ns.

Here, a chambered coverglass containing Coumarin 6 is illuminated with a 440 nm laser at sufficient power to achieve approximately 100,000 counts per second in the donor emission channel; in this case, a 480/40 nm band pass filter. Frame averaging is used to accumulate approximately 100 peak counts per pixel. It is important to accumulate at least 100 peak counts to have sufficient signal to assign lifetimes to each image pixel. The phasor plot is generated directly from the frequency characteristics of the emission signals, and it displays the distribution of the lifetimes for all the pixels in the image (Figure 4). Note that the lifetime distribution for Coumarin 6 is centered on the universal semicircle, indicating a single-component mean lifetime (τ_m_) of 2.5 ns. To verify that the system is accurately calibrated, measurements are then made of a second dye, HPTS (8-Hydroxypyrene-1,3,6- trisulfonic acid) dissolved in phosphate buffer. Again, the 440 nm laser power was adjusted to achieve approximately 100,000 counts per second in the donor emission channel, and frame averaging is used to accumulate approximately 100 peak counts per pixel. The phasor plot for HPTS clearly shows that the lifetime distribution is shifted to the left along the semicircle relative to Coumarin 6 (Figure 4). The distribution falls directly on the semicircle, indicating a single-component mean lifetime of 5.3 ns.

### 3.3. Testing the FLIM System Using “FRET Standard” Proteins

Before making FLIM measurements from the experimental samples expressing the FP-based biosensor proteins, it is important to test both the experimental system and the microscope system using probes with known FRET efficiencies. The “FRET standard” approach developed by the Vogel laboratory provides a straightforward approach to achieve this goal [11,34]. Here, we generated plasmid vectors encoding an improved variant of CFP, mCerulean3, coupled to the optimized YFP variant mVenus. The mCerulean3 has reduced photoswitching behavior and improved photostability compared to other CFPs [35]. When the plasmid is transfected into cells, a fusion protein is produced that contains mCerulean3 directly coupled to mVenus through the short five amino acid (5aa) linker-SGLRS-. Since the fluorescence lifetime can be very sensitive to the local environment of the donor fluorophore, we also generated a similar plasmid that encodes the mCerulean3 linked to mutant variant of mVenus, called Amber. A mutation in Amber converts the chromophore tyrosine to a cysteine producing a non-fluorescent form of Venus that folds correctly, but does not act as a FRET acceptor [11]. Thus, while the local environment surrounding the donor fluorophore is very similar for the Cerulean3-5aa-Amber and Cerulean3-5aa-Venus fusion proteins, there is no energy transfer to Amber, so the donor remains unquenched. In addition, a plasmid encoding mCerulean3 linked to mVenus through the 229-amino acid tumor necrosis factor receptor associated factor (TRAF) domain (Cerulean3-TRAF-Venus) was also generated, and this serves as a low FRET efficiency standard [11].

**Figure 5:**
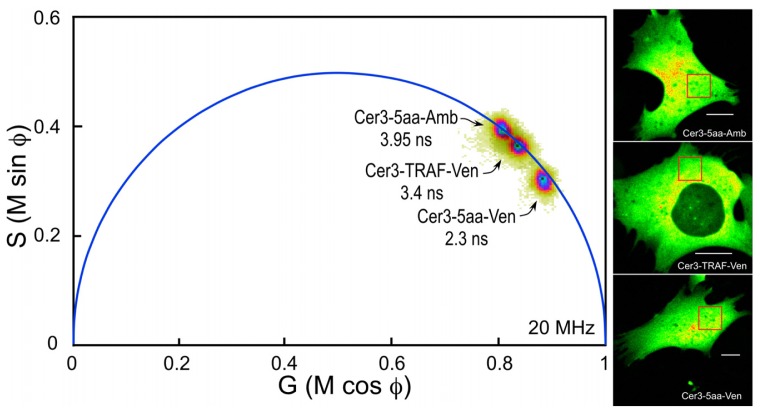
The phasor plot analysis of the lifetime for the Cerulean3 Förster resonance energy transfer (FRET) standards. Cells expressing Cerulean3-5aa-Amber (unquenched donor), Cerulean3-TRAF-Venus, or Cerulean3-5aa-Venus were imaged by FLIM (see text for details). The intensity image for each of the representative cells is shown in the right panels, and the calibration bars indicate 10 μm. The lifetime distribution for all pixels in each of the background-subtracted images is displayed on the polar plot. The average lifetime for each of the linked probes was determined in the region of interest (ROI) indicated by the red box in each image.

The composite phasor plot in Figure 5 compares the unquenched and quenched donor lifetimes for Cerulean3 in the FRET standard fusion proteins expressed in living cells. Note that the lifetime distribution for Cerulean3-5aa-Amber (the unquenched donor) falls on the semicircle, indicating that it best fits a single-component decay. The average lifetime, determined for the region of interest (ROI) shown in the intensity image, was 3.95 ns. In contrast, the lifetime distributions for the cells expressing Cerulean3-TRAF-Venus and Cerulean3-5aa-Venus fall slightly inside the universal semicircle on the phasor plot, indicating a heterogeneous distribution of lifetimes (Figure 5). The average donor lifetime for the fusion protein in which Cerulean3 is separated from Venus by the large TRAF domain, determined for the indicated ROI, is shorter than that of the unquenched donor (3.4 ns compared to 3.95 ns), which is consistent with a low efficiency of energy transfer. The FRET standard with the short linker, Cerulean3-5aa-Venus, has a donor lifetime that is significantly more quenched because of the close proximity of the acceptor, with an average lifetime in the indicated ROI of 2.3 ns.

The measurement of the shorter donor fluorescence lifetime that results from energy transfer to an acceptor is the most direct method for quantifying FRET. The efficiency of energy transfer (*E*_FRET_) is simply determined from the ratio of the donor lifetime in the presence (τ_DA_) and absence (τ_D_) of acceptor (Equation 1):

(1) $$E_{FRET}=1-\frac{\tau_{DA}}{\tau_{D}}$$

Here, the unquenched donor lifetime (τ_D_) was determined from a population of cells that expressed Cerulean3-5aa-Amber. The results (Table 1) demonstrate that the mean lifetime (τ_m_) for the unquenched donor (Cerulean3-5aa-Amber) was 3.93 ns (*n* = 21 cells). The donor lifetime in the presence of the acceptor (τ_DA_) was then determined for populations of cells expressing the linked donor-acceptor fusion proteins (Table 1). In sharp contrast to the unquenched donor the τ_m_ for the Cerulean3-5aa-Venus fusion protein was significantly reduced to 2.37 ns (*n* = 19 cells), corresponding to an average FRET efficiency of 39.8%. With the much longer linker separating donor and acceptor in the Cerulean3-TRAF-Venus fusion protein, there was substantially less donor quenching, with a τ_m_ of 3.65 ns (*n* = 20 cells), corresponding to mean FRET efficiency of 7.1%. The FRET standard approach enables robust validation of the experimental system used to measure biosensor probe activities.

## 4. Measuring Biosensor Probe activity in Living Cells

**Figure 6:**
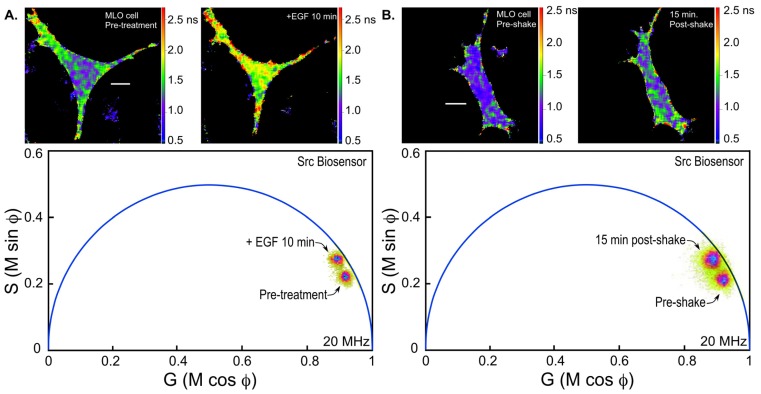
The phasor plot analysis of the lifetime for the Src biosensor in MLO-Y4 cells. (**A**) A representative cell expressing the Src biosensor was imaged by FLIM (see text for details). The polar plot indicates the lifetime distribution for the cell prior to epidermal growth factor (EGF) treatment (pre-treatment), and the lifetime map (top panel) shows the average lifetimes throughout the cell displayed with a look-up table that uses cool to warm colors for shorter to longer lifetimes; the calibration bar indicates 10 μm. EGF (50 μg/mL) was added to the culture medium, and the same cell was imaged by FLIM after 10 min. The shift in the lifetime distribution on the polar plot indicates the opening of the Src biosensor probe in response to EGF, resulting in an increase in probe lifetime corresponding to decreased FRET (see Figure 1). The lifetime map (top panel) shows the cellular regions with increased probe lifetime; (**B**) A cell expressing the Src biosensor is shown before (left panel) and after mechanical shaking (right panel; see text for details). The polar plot indicates the lifetime distribution for the cell before (pre-shake) and after mechanical shaking (20 min post-shake), and the lifetime maps in the top panels show the average lifetimes throughout the cell.

Here, FLIM measurements of biosensor protein activities in living cells are illustrated using the Src (Figure 1) and cAMP Epac (Figure 2) biosensor probes. Mechanical stimuli activate integrins and the cytoskeleton, and trigger cell-signaling events involving the Src kinase that regulate cell function in various tissues including the skeleton [36]. We are using the Src biosensor probe to investigate the response of living bone cells to a mechanical stimulus applied by dynamic fluid flow over the cell surface. First, to demonstrate the Src probe response, Src activity in MLO-Y4 osteocyte-like cells expressing the Src biosensor was increased by treatment with epidermal growth factor (EGF) for 10 min (Figure 6A). Note that the phasor distribution for the Src biosensor probe falls inside the universal semicircle, indicating a heterogeneous distribution of lifetimes because of FRET from the probe in the closed conformation. Using FLIM analysis, three ROI (membrane, cytoplasm and nucleus) were evaluated for changes in Src activity. All three ROIs showed a significant increase in Src activity in response to 10 min of EGF treatment as measured by an increase in Src biosensor lifetime (data not shown). Further, as would be predicted from an agonist like EGF, which binds to and activates a receptor at the plasma membrane and interacts with Src, the greatest increase in Src biosensor lifetime was generally observed adjacent to or near the plasma membrane. In contrast, the molecular mechanisms through which mechanical stimulation of cells by fluid shear stress induced Src activity are less clear. Previous studies showed that mechanical stimulation of cells by fluid shear increases Src activation (as measured by western blotting of tyrosine phosphorylated Src) via an integrin-dependent mechanism [37,38]. The Src biosensor offers a unique opportunity to determine the spatial distribution of Src activity in response to fluid flow that has not previously been possible. Here, MLO-Y4 cells were exposed to 5 min of fluid shear on an orbital shaking platform that generates a fluid flow shear rate between 15–25 dynes/cm^2^ (Figure 6B). The Src biosensor lifetimes determined 20 min after fluid flow were significantly elevated throughout the cell. Compared to cells stimulated with EGF (panel A) Src activity in cells stimulated by fluid shear appeared higher both near the membrane and in the cell interior. ROI analysis of MLO-Y4 cells subjected to fluid shear stress suggest that, unlike cells stimulated with EGF, mechanical stimulation rapidly increases Src activity in and around the nucleus (manuscript under review). This raises the intriguing possibility that mechanical signals detected at the cell’s surface via integrins may be rapidly transmitted to the nucleus via a mechanism that involves increased nuclear Src activity.

**Figure 7:**
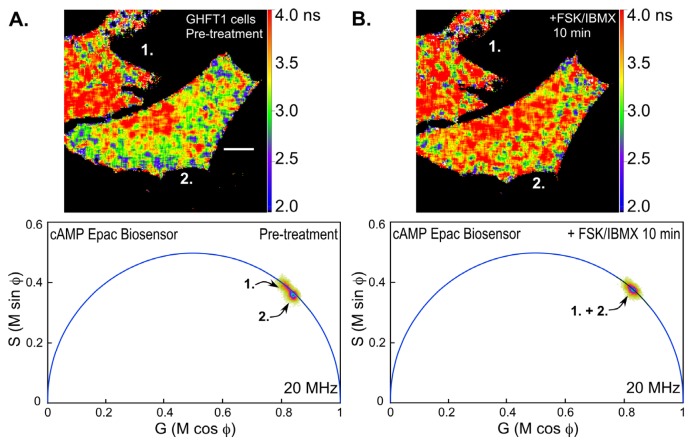
Changes in the lifetime of the cAMP biosensor T_EPAC_VV in response to forskolin treatment. (**A**) The polar plot for two mouse pituitary GHFT1 cells expressing T_EPAC_VV biosensor prior to treatment with forskolin (FSK)/IBMX (pre-treatment, see text for details) is shown, indicating discrete lifetime distributions corresponding cell 1 and cell 2. The lifetime map (top panel) shows the average lifetimes throughout the cells; the calibration bar indicates 10 μm; (**B**) A mixture of FSK and IBMX was added to the culture, and the same cells were imaged by FLIM after 10 min. The shift in the lifetime distribution on the polar plot indicates the opening of the cAMP Epac biosensor probe in response to the treatment, resulting in an increase in probe lifetime in cell 2 (top panel).

Next, we examined changes in intracellular cAMP levels using the Epac-based biosensor probe. Mouse pituitary GHFT-1 cells expressing the T_EPAC_VV biosensor were challenged with a mixture of forskolin (FSK, 25 μM), a diterpene activator of adenylyl cyclase, and IBMX (3-isobutyl-1-methylxanthine, 100 μM) a phosphodiesterase inhibitor, and changes in the biosensor signal were monitored by FLIM (Figure 7). Notice that the polar plot from the FLIM image of the cells prior to treatment with FSK/IBMX (pre-treatment) clearly indicates two separate lifetime distributions corresponding to the indicated cells (cell 1 and cell 2, Figure 7A). The lifetime map (top panel) shows the average lifetimes throughout the cells, demonstrating that cell 1 has a longer average lifetime, corresponding reduced donor quenching and activation of the Epac biosensor. This most likely indicates that the biosensor proteins were already activated in cell 1 relative to cell 2. The FLIM image of the same cells was then reacquired 10 min following treatment with FSK/IBMX (Figure 7B). The polar plot from the post-treatment FLIM image now shows that the lifetime distributions for cell 1 and cell 2 have merged. The lifetime map (top panel) shows that both cells now have similar average lifetimes. This is consistent with the activation of the biosensor probes in cell 2 following the treatment.

## 5. Conclusions

In contrast to biochemical assays, which typically are end-point assays performed on populations of cells, live-cell microscopy can monitor the spatial and temporal regulation of cell signaling events inside single cells. In this regard, live-cell imaging of changes in biosensor protein activities over time can provide a unique perspective on the regulation of cell signaling events. That is not to say, however, that the biosensor approach is without limitations. Analogous to the Heisenberg uncertainty principle in physics, every measurement perturbs the system that is being measured. For most biosensor probes, the sensor unit is a target for modification by the cell signaling machinery, and does not form a covalent complex with cellular proteins. However, the transfection approaches for introducing FP-biosensor probes can yield very high levels of the fusion proteins in the target cells, especially when strong promoters are used. The high-level expression of these probes can lead to sequestration of the endogenous effector molecules and result in aberrant signals in inappropriate subcellular domains [39]. Although the FRET measurements from biosensor probes, when collected and quantified properly, are remarkably robust, there can be substantial heterogeneity in the measurements. As was shown here (Table 1), the data must be collected from multiple cells and statistically analyzed to prevent the user from reaching erroneous conclusions from a non-representative measurement.

Fluorescence lifetime is exquisitely sensitive to the probe environment, but because the measurements are made in the time domain, they are insensitive to confounding factors that commonly limit measurements by intensity-based imaging. For example, FLIM measurements are not adversely affected by variations in probe concentration (either regional intracellular variation or cell-to-cell variations), excitation intensity, or light scattering. Furthermore, since the fluorescence lifetime of a fluorophore is sensitive to its environment, FLIM can be a superior choice for detecting signal changes from biosensor probes that report ion binding, pH, or protein phosphorylation. As demonstrated here, the genetically encoded FRET standard proteins (Section 3.3) are useful tools for optimizing experimental culture conditions for FRET measurements, and for evaluating imaging systems for the detection of FRET. The low FRET efficiency standards are especially useful for assessing the background noise in the system.

In this regard, it is critical to identify the sources of noise in FRET-FLIM measurements to determine the reliability of the data analysis. For instance, a donor fluorophore whose intrinsic lifetime has multiple components may not be suitable for FLIM-FRET, since it will complicate the data analysis [12]. Although the analysis of FLIM data has become routine with the advanced software that is available, an understanding of the physics that underlies the changes in fluorescence lifetime is necessary for processing of the FLIM data and the interpretation of the results [10]. Finally, the major limitation of the FLIM approach for detecting biosensor probe activities is that the acquisition of FRET-FLIM data is typically slow relative to other methods. For example, ratiometric methods can measure changes in biosensor activities over the time frame of seconds, while FLIM methods require 10’s of seconds to obtain a single measurement with high accuracy. The studies describe here using the FD FLIM method required about 45 s to acquire sufficient photon counts to accurately assign lifetimes, which limit its application for monitoring very dynamic events. There are FLIM methods based on wide-field imaging using a gated image intensifier camera, or spinning disk confocal systems that have the ability to acquire data in seconds [10,40]. As the technology continues to evolve, it is expected that the time required to obtain FLIM measurements will decrease significantly, making this technique even more useful for measurements of dynamic changes in biosensor activities in single living cells. What is more, since lifetime measurements are made only in the donor channel, this approach has great potential for the simultaneous monitoring of multiple intracellular signaling events through the combination of two different FRET-based biosensor proteins [41,42].

## Figures and Tables

**Table 1:** FLIM-FRET measurements of the Cerulean3 fusion proteins expressed in living cells.

Fusion protein	τ_m_ (ns) a	*E*_FRET_^b^
Cerulean3-5aa-Amber (*n* = 21)	3.93 ± 0.10	NA
Cerulean3-5aa-Venus (*n* = 19)	2.36 ± 0.074	39.8 ± 1.9%
Cerulean3-TRAF-Venus (*n* = 20)	3.65 ± 0.088	7.1 ± 2.2%

a ±SD;

b Determined by *E* = 1 − (τ_DA_/τ_D_); see text.
